# Temporal relationships between happiness and psychiatric disorders and their symptom severity in a large cohort study: the Netherlands Study of Depression and Anxiety (NESDA)

**DOI:** 10.1186/s12888-021-03346-4

**Published:** 2021-07-10

**Authors:** Philip Spinhoven, Bernet M. Elzinga, Brenda W. J. H. Penninx, Erik J. Giltay

**Affiliations:** 1 Institute of Psychology, Leiden University, Wassenaarseweg 52, 2333 AK Leiden, the Netherlands; 2 Department of Psychiatry, Leiden University Medical Center, Leiden, the Netherlands; 3 Department of Psychiatry, Amsterdam University Medical Center, Amsterdam, the Netherlands

**Keywords:** Happiness, Depression, Social anxiety disorder, Prediction, Longitudinal

## Abstract

**Background:**

Notwithstanding the firmly established cross-sectional association of happiness with psychiatric disorders and their symptom severity, little is known about their temporal relationships. The goal of the present study was to investigate whether happiness is predictive of subsequent psychiatric disorders and symptom severity (and vice versa). Moreover, it was examined whether changes in happiness co-occur with changes in psychiatric disorder status and symptom severity.

**Methods:**

In the Netherlands Study of Depression and Anxiety (NESDA), happiness (SRH: Self-Rated Happiness scale), depressive and social anxiety disorder (CIDI: Composite Interview Diagnostic Instrument) and depressive and anxiety symptom severity (IDS: Inventory of Depressive Symptomatology; BAI: Beck Anxiety Inventory; and FQ: Fear Questionnaire) were measured in 1816 adults over a three-year period. Moreover, we focused on occurrence and remittance of 6-month recency Major Depressive Disorder (MDD) and Social Anxiety Disorders (SAD) as the two disorders most intertwined with subjective happiness.

**Results:**

Interindividual differences in happiness were quite stable (ICC of .64). Higher levels of happiness predicted recovery from depression (OR = 1.41; 95% CI = 1.10–1.80), but not social anxiety disorder (OR = 1.31; 95%CI = .94–1.81), as well as non-occurrence of depression (OR = 2.41; 95%CI = 1.98–2.94) and SAD (OR = 2.93; 95%CI = 2.29–3.77) in participants without MDD, respectively SAD at baseline. Higher levels of happiness also predicted a reduction of IDS depression (sr = − 0.08; 95%CI = -0.10 - -0.04), and BAI (sr = − 0.09; 95%CI = -0.12 - -0.05) and FQ (sr = − 0.06; 95%CI = -0.09 - -0.04) anxiety symptom scores. Conversely, presence of affective disorders, as well as higher depression and anxiety symptom severity at baseline predicted a subsequent reduction of self-reported happiness (with marginal to small sr values varying between −.04 (presence of SAD) to −.17 (depression severity on the IDS)). Moreover, changes in happiness were associated with changes in psychiatric disorders and their symptom severity, in particular with depression severity on the IDS (sr = − 0.46; 95%CI = −.50 - -.42).

**Conclusions:**

Results support the view of rather stable interindividual differences in subjective happiness, although level of happiness is inversely associated with changes in psychiatric disorders and their symptom severity, in particular depressive disorder and depression severity.

## Background

As the title ‘No health without mental health’ of the pivotal paper of Prince and colleagues [1] suggests, health is more than the absence of somatic disease and in accordance with the World Health Organization definition of health from 1948 also comprises ‘a state of complete physical, mental and social well-being’. Life satisfaction and happiness are indicators of subjective well-being and constitute an important dimension of positive mental health in addition to e.g. emotional and social intelligence and the ability to work and to love [2].

Previous studies have found that levels of life satisfaction and happiness are higher among persons with a higher socio-economic status, a stable intimate relationship, social resources and support, work, financial resources, good somatic health, and the personality characteristics of low neuroticism and high extraversion, although the amount of variance explained is quite small [3, 4].

The set-point theory of happiness suggests that happiness is determined primarily by heredity and by the personality traits of neuroticism and extraversion ingrained in us early in life, and as a result happiness remains relatively constant throughout our lives [5, 6]. According to this theory, level of happiness may change transiently in response to life events, but will return to its baseline level as a consequence of habituation to those events and their consequences over time [7].

As mental health problems are the strongest predictor of life satisfaction and happiness [8], the question arises to what extent the level of happiness not only changes in response to external events but also in response to suffering from a mental health problem. Notwithstanding the firmly established association of mental health problems with happiness, most studies into this relationship cannot adequately address this issue because of (a) diverging ways of measuring mental health problems ranging from self-reported distress to interview-based psychiatric assessments; (b) cross-sectional designs only assessing the relationship of mental health problems with happiness at one point in time; and (c) analysis of the effects of happiness on better life outcomes including mental health without consideration of a reverse association.

In a previous clinical cohort study on happiness in depression and anxiety disorder, particularly persons with more depressive symptoms, major depressive disorder (MDD), social anxiety disorder (SAD) and comorbid emotional disorders reported lower levels of happiness, while level of happiness was less strongly related to panic disorder, generalized anxiety disorder and agoraphobia [9]. A serious study limitation was that happiness was only measured once. Because happiness now has also been measured in a subsequent wave after 3 years in this cohort study, this provides the opportunity to examine (the course of) happiness in anxiety and depression longitudinally and to examine in more detail the temporal relationships of (changes in) happiness with (changes in) psychiatric disorders and their symptom severity. On the basis of our previous study, we will focus on 6-month recency MDD and SAD as the two disorders most intertwined with subjective happiness, as well as severity of depressive and anxiety symptoms.

In the present study we will not only investigate the prospective value of happiness for psychiatric disorders and their symptom severity, but also the reverse association. Moreover, we will investigate the association of changes in happiness with changes in psychiatric disorders and their symptom severity. We expected relatively stable interindividual differences in level of happiness (i.e., relative stability), but also that changes in psychiatric disorders and their symptom severity are associated with changes in subjective levels of happiness (i.e., no absolute stability). Moreover, we expected prospective relationships of happiness with future psychiatric disorders and their symptom severity, but also the reverse relationship.

## Method

### Study design and participants

The Netherlands Study of Depression and Anxiety (NESDA) is a multi-site naturalistic ongoing cohort study developed to investigate antecedents, course, and consequences of depression and anxiety disorders. A total of 2981 persons aged 18 to 65 years were included, recruited from the general population (*n* = 564), primary care (*n* = 1610), and mental health organizations (*n* = 807). The NESDA sample consists of healthy controls, persons with a prior history of depression and/or anxiety disorders, and persons with a current depression and/or anxiety disorder. General exclusion criteria were a primary diagnosis of severe psychiatric disorders such as a psychotic disorder, obsessive-compulsive disorder, bipolar disorder, or severe addiction disorder, and not being fluent in Dutch. More information about the framework of the NESDA study can be found somewhere else (see [10]). The study protocol of NESDA was approved by the Ethical Committees of the participating universities and written informed consent was obtained from all respondents. The framework of this longitudinal cohort study with repeated measurements of core sociodemographic and clinical variables (psychiatric diagnoses and symptom severity) allows to introduce new study variables to assess their concurrent and prospective relationships with other data collected in the NESDA study.

The baseline assessment consisted of an assessment of demographic and personal characteristics, a standardized diagnostic psychiatric interview, and a medical assessment including blood sampling. After 2, 4, 6, and 9 years, a face-to-face follow-up assessment was performed with a response rate of 75.7% (*n* = 2256) after six, and of 69.4% (*n* = 2069) after 9 years. The Self-Rating of Happiness scale (SRH) was administered for the first time after 6 years (i.e., the baseline of the present study) and completed by 2142 of 2256 participants (95.0%). Of these 2142 participants, 1816 participants (attrition rate = 15.3%) completed the SRH also after 9 years (i.e., the follow-up in the present study), constituting our present sample.

We created seven partly overlapping subgroups: (a) persons with no current depressive or anxiety disorder at baseline and follow-up (unaffected groups, *n* = 1126); (b) persons with no MDD or (c) SAD at baseline but with MDD resp. SAD at follow-up (occurrence groups, *n* = 161 and *n* = 86, respectively); (d) persons with MDD or (e) SAD at baseline and no MDD respectively SAD at follow-up (recovery groups, *n* = 166 and *n* = 72, respectively); (f) persons with MDD or (g) SAD at baseline and follow-up (chronically affected groups, *n* = 94 and *n* = 86, respectively). As the group of participants with both MDD and SAD (*n* = 63 both at baseline and follow-up including 25 participants with both MDD and SAD at both time points) was too small to analyze separately, these participants were included in multiple groups resulting in partially overlapping subgroups.

### Measures

#### Self-Rated Happiness (SRH)

In order to measure the degree of happiness at baseline and follow-up, we used the Self-Rating of Happiness scale (SRH) [11]. The SRH is a single-item self-rating scale that asks about happiness as a personal characteristic (not specifically related to certain events). The SRH consists of the following question: “How happy or unhappy do you feel with your life in general?”. Following this question, a series of numbers from 1 to 7 with corresponding labels was written vertically: 1 ‘completely happy’; 2 ‘very happy’; 3 ‘quite happy’; 4 ‘neither happy, nor unhappy’; 5 ‘quite unhappy’; 6 ‘very unhappy’; and 7 ‘completely unhappy’. Measuring happiness by a single item is reliable, valid, and viable in community surveys as well as in cross-cultural comparison [12]. The SRH was reverse scored, so that higher scores denote a higher degree of happiness.

#### Psychiatric diagnosis

DSM-IV defined 6-month recency depressive (i.e., major depressive disorder, dysthymia) and anxiety (i.e., panic disorder with or without agoraphobia, social anxiety disorder, generalized anxiety disorder, agoraphobia without panic) disorders were assessed using the Composite Interview Diagnostic Instrument (CIDI, version 2.1) at baseline and follow-up. The CIDI is a fully standardized diagnostic interview, that is used worldwide to classify psychiatric diagnoses according to DSM-IV criteria [13]. It has shown high interrater reliability, high test-retest reliability and high validity for depressive and anxiety disorders [14].

#### Symptom severity

Severity of depression symptoms was measured with the 30-item Inventory of Depressive Symptomatology self-report version (IDS-SR) [15] at baseline and follow-up, which has shown high correlations with observer-rated scales such as the Hamilton Depression Scale [16]. Severity of anxiety was measured using the 21-item Beck Anxiety Inventory (BAI) [17, 18] at baseline and follow-up. This scale has shown sound psychometric properties such as factorial validity, internal consistency, and test-retest stability, as well as adequate convergent and discriminant validity. Severity of avoidance symptoms was measured using the 15-item self-report Fear Questionnaire (FQ) [19]. The Dutch translation of the FQ has good psychometric properties [20].

### Statistical analyses

First, in order to evaluate changes in self-reported happiness, depression and anxiety severity during the follow-up period, baseline and 3-yr follow-up SRH, IDS, BAI and FQ scores were compared using dependent t-tests within the total sample and the seven diagnostic status subgroups and the magnitude of the within group differences was evaluated by calculating Cohen’s d [21]. A *d* of .2, .5, and .8 was considered as a small, medium, and large effect size, respectively. Intra-class correlation coefficients (ICCs: 1-way random effects model with single measure reliability) were calculated to determine stability of SRH scores. ICC values of .40, .60, and .75 were deemed fair, good, and excellent, respectively [22].

The predictive value of baseline SRH scores for the occurrence or recovery of MDD and SAD was examined with separate logistic regression analyses with baseline SRH scores as independent variable and occurrence or recovery of MDD or SAD as dependent variable. The other temporal relationships of SRH scores with psychiatric diagnoses and symptom severity were examined using multiple regression analyses: (a) in order to determine the effect of self-reported happiness at baseline on changes in depression and anxiety severity, IDS, BAI and FQ scores at follow-up were regressed on baseline SRH scores while controlling for baseline IDS, BAI respectively FQ scores; (b) in order to analyze to what extent psychiatric disorder and symptom severity at baseline predicted changes in SRH scores, SRH scores at follow-up were regressed on presence of MDD and SAD at baseline, respectively IDS, BAI, and FQ scores at baseline in separate analyses, while controlling for SRH baseline scores; (c) in order to analyze the association of changes in self-reported happiness with changes in symptom severity, separate analyses were performed with SRH scores at follow-up as dependent variable and residualized (i.e., follow-up scores corrected for baseline scores) change scores on the IDS, BAI, and FQ as dependent variables, while controlling for baseline SRH scores; and (d) in order to analyze the relation of changes in diagnostic status with changes in SRH, SRH scores at follow-up were regressed on the occurrence or recovery of MDD and SAD, while controlling for baseline SRH scores in separate analyses.

For the multiple regression analyses, semi-partial correlation coefficients (sr) for each predictor variable are provided as measures for the strength of the association of predictor variables with outcome over and above control variables. A semi-partial correlation coefficient represents the correlation between the criterion and a predictor that has been residualized with respect to all other predictors in the eq. A sr of .1, .3, and .5 was considered as a small, medium, and large effect size, respectively [21].

In all the analyses above we controlled for age, gender and years of education as possible confounding variables. Statistical analyses were run using SPSS version 26 [23] and a significance level of *p* < .05 was used for all analyses.

## Results

### Sample characteristics

Table 1 shows the sociodemographic, clinical, and psychological characteristics of the total sample at baseline and follow-up. Of note is that, despite the fact that this sample contains quite many persons with current and/or remitted emotional disorders, at baseline and follow-up around three-quarter of the participants indicated to be ‘completely happy’ to ‘quite happy’, while less than 10% of the participants indicated that they felt ‘quite unhappy’ to ‘very unhappy’.

**Table 1:** Sociodemographic, clinical, and psychological characteristics of the total sample at baseline (*n* = 1816)

	Baseline		3-yr follow-up	
Variables	M / n	SD / %	M / n	SD / %
*Sociodemographic characteristics*				
Age	48.3	13.1		
Female gender, *n* (%)	1198	66.0		
Years of education	13.0	3.3		
*Clinical characteristics*				
IDS	14.8	11.5	14.6	11.4
BAI	8.1	8.2	7.5	8.2
FQ	16.9	16.9	15.8	16.8
Current depression (CIDI), *n* (%)	260	14.3	255	14.0
Current SAD (CIDI), *n* (%)	149	8.2	155	8.5
*Psychological characteristic*				
SRH	5.0	1.0	5.1	1.0
‘completely happy’ to ‘quite happy’ (5–7)	1373	75.6	1412	77.8
‘neither happy, nor unhappy’ (4)	301	16.6	258	14.2
‘quite unhappy’ to ‘very unhappy’ (1–3)	142	7.8	146	8.0

*IDS* Inventory of Depressive Symptomatology, *BAI* Beck Anxiety Inventory, *FQ* Fear Questionnaire, *CIDI* Composite Interview Diagnostic Instrument, *SRH* Self-Rated Happiness scale

### Changes in self-reported happiness and psychiatric disorders and their symptom severity between baseline and follow-up

In the total group, SRH scores at baseline were strongly related to SRH scores at follow-up (*r* = .65, *p* < .001), while the increase between baseline and follow-up scores although statistically significant (t (1815) = 3.17, *p* < .001) had a negligible effect size (*d* = .08). The ICC of .64 (95%CI: .62–.67) indicates good stability of SRH scores (see Table 2).

**Table 2:** Baseline and 3-yr follow-up SRH scores in the different MDD and SAD groups

Groups	*n*	SRH at baseline		SRH at 3-yr follow-up		Results paired t-tests		Stability SRH scores	
		*M*	SD	*M*	SD	t	d	ICC	95%CI
Total sample	1816	5.0	1.0	5.1	1.0	3.17 **	0.12	.64	.62–.67
Unaffected during FU	1126	5.4	0.8	5.4	0.8	3.71 ***	0.11	.57	.53–.60
Occurrence during FU									
MDD	161	4.7	0.9	4.3	1.1	−5.18 ***	0.37	.54	.39–.67
SAD	86	4.5	1.0	4.4	1.2	−0.70 ns	0.08	.52	.34–.65
Recovery during FU									
MDD	166	4.1	1.0	4.6	1.0	6.29 ***	.56	.57	.53–.60
SAD	72	4.3	1.0	4.7	1.0	3.57 ***	.43	.63	.47–.75
Chronically affected during FU									
MDD	94	3.8	1.2	3.8	1.2	0.27 ns	0.00	.54	.39–.67
SAD	69	4.0	1.1	4.1	1.2	1.13 ns	0.14	.73	.60–.82

*SRH* Self-Rated Happiness scale. The *t* values are results of the paired sample *t*-test on SRH scores at baseline and 3-yr follow-up, d = Cohen’s d; *ICC* Intra-Class Correlation

**Fig. 1:**
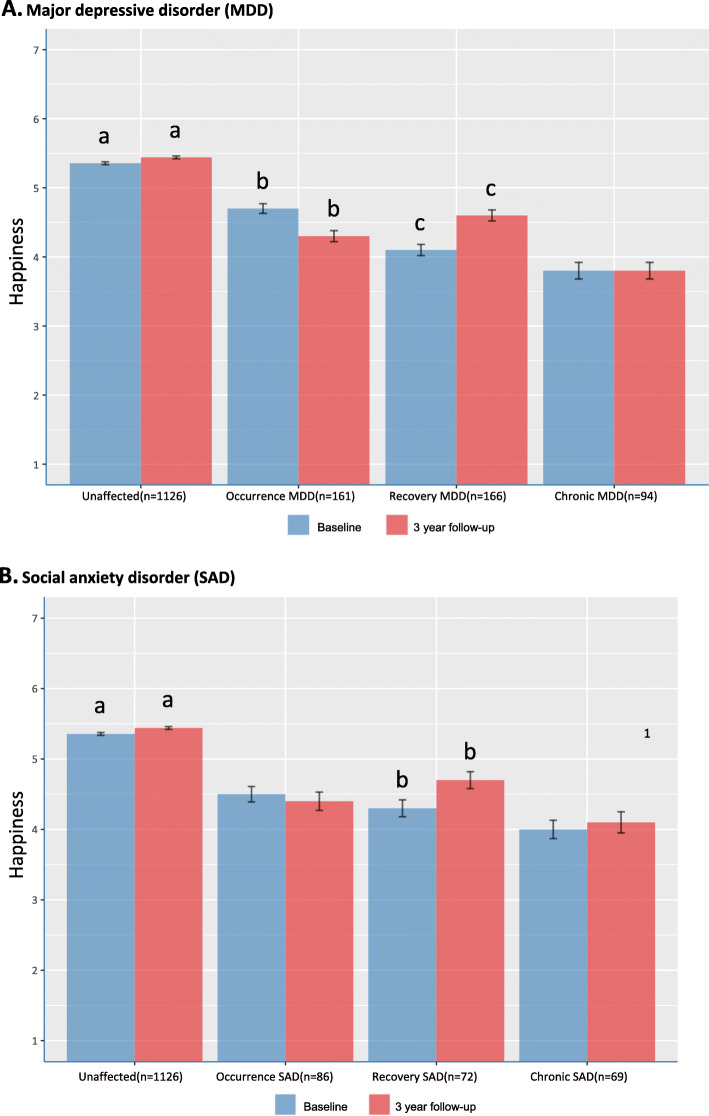
Self-rated happiness scores in the different MDD and SAD groups at baseline and 3-year follow-up. Note. Bars with the same letter are statistically different according to paired t-test

The rate of occurrence of MDD at follow-up in participants with no MDD at baseline was 10.3% (161/1556) and of SAD at follow-up in participants with no SAD at baseline 5.1% (86/1675). The rate of recovery of MDD at follow-up in participants with MDD at baseline was 63.8% (166/260) and of SAD at follow-up in participants with SAD at baseline 51.1% (72/141). Figure 1 A and B present baseline and follow-up SRH scores in the different MDD respectively SAD groups. As can be seen SRH scores at baseline and follow-up were highest in the unaffected group and lowest in the chronically affected subgroups. The increase of SRH scores in the unaffected group had a negligible effect size. Decreases in SRH scores were significant with a moderate effect size in the MDD recovery group and with a small effect size in the SAD recovery group. The significant increase in SRH scores had a small effect size in the MDD occurrence group and negligible effect size in de SAD occurrence group. Changes in SRH scores in both chronically affected groups were non-significant. ICCs in the different subgroups varied from .52 to .73 indicating fair to good stability of SRH scores across subgroups (see Table 2).

BDI scores at baseline did not differ significantly from corresponding follow-up scores (t (1814) = 0.89, *p* = .37), BAI scores at baseline showed a significant decline at follow-up (t (1806) = 4.62, *p* < .001), although with a negligible effect size (d = .01). The same applied to the slight decrease of FQ scores from baseline to follow-up (t (1798) = 4.41, *p* < .001; d = .10).

### Prediction of changes in psychiatric disorders and their symptom severity by self-reported happiness at baseline

Logistic regression analyses showed that higher levels of self-reported happiness in persons with no MDD or SAD at baseline were predictive of non-occurrence of MDD (OR = 2.41; 95%CI = 1.98–2.94), as well as non-occurrence of SAD (OR = 2.93; 95%CI = 2.29–3.77), while higher levels of self-reported happiness in persons with MDD at baseline were predictive of recovery of MDD (OR = 1.41; 95% CI = 1.10–1.80), but not of SAD (OR = 1.31; 95%CI = .94–1.81). Repeating these analyses while additionally controlling for symptom severity at baseline (IDS, BAI and FQ scores) showed that although the predictive power of SRH scores was attenuated, the level of happiness remained predictive of non-occurrence of MDD (OR = 1.45;95%CI = 1.14–1.84), respectively of SAD (OR = 1.57; 95%CI = 1.13–2.18) in participants with no MDD, respectively SAD at baseline.

Subsequent multiple regression analyses with baseline SRH scores as independent variable and corresponding baseline symptom severity scores as control variables, showed that higher levels of happiness also predicted a reduction of IDS depression (sr = − 0.08; 95%CI = -0.10 - -0.04), and BAI (sr = − 0.09; 95%CI = -0.12 - -0.05) and FQ (sr = − 0.06; 95%CI = -0.09- -0.04) anxiety symptom scores, but that the amount of additionally explained variance in symptom reduction was negligible (sr = < .10).

### Prediction of changes in self-reported happiness by psychiatric disorders and their symptom severity at baseline

Separate multiple regression analyses with follow-up SRH scores as dependent variable and presence of MDD or SAD and IDS, BAI or FQ scores at baseline as independent variables, while controlling for baseline SRH scores showed that each of these variables significantly predicted a subsequent reduction of self-reported happiness (with marginal to small sr values varying between −.04 (presence of SAD at baseline) to −.17 (depression severity on the IDS)) (see Table 3).

**Table 3:** Results of separate multiple regression analyses with presence of MDD and SAD and IDS, BAI and FQ scores at baseline as independent variable and SRH scores at 3-yr follow-up as dependent variable

	n	Beta	t	p	Semipartial correlation	95%CI
MDD	1816	−.06	−3.00	.003	−.05	−0.09 - -0.02
SAD	1816	−.04	−1.96	.05	−.04	−0.07 - - 0.00
IDS	1815	−.23	−9.50	< .001	−.17	−0.20 - - 0.13
BAI	1812	−.13	−6.15	<.001	−.11	−0.14 - - 0.07
FQ	1793	−.09	−4.58	<.001	−.08	−0.12 - -0.05

All regression analyses controlled for age, gender, education and SRH scores at baseline

### Association between changes in self-reported happiness and changes in psychiatric disorders and their symptom severity

**Fig. 2:**
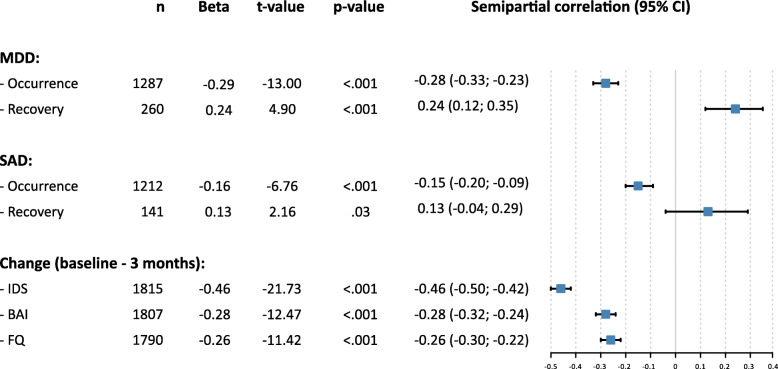
Results of separate multiple regression analysis with occurrence and recovery of MDD or SAD and changes on IDS, BAI and FQ during 3-yr follow-up as independent variable and corresponding changes in self-reported happiness (SRH) as dependent variable. Note. All regression analyses controlled for age, gender, education and SRH scores at baseline

As shown in Fig. 2 the occurrence of MDD and SAD was significantly associated by lower SRH scores at follow-up, while the recovery of both MDD but not SAD had the opposite effect. Moreover, changes in IDS, BAI and FQ were significantly associated with changes in SRH scores, with higher symptom severity being associated with lower SRH scores. Effect sizes were small, except for the moderate effect sizes of changes in depression severity with changes in SRH scores (sr = − 0.46; 95%CI = −.50 - -.42).

## Discussion

This study investigated temporal relationships between self-reported happiness and psychiatric diagnoses and symptom severity using data of 1816 participants from a longitudinal clinical cohort study. In our total sample including many subjects who had or have had emotional disorders, threequarters of the participants reported to be quite happy to completely happy, while less than 10% indicated to be quite unhappy to very unhappy. Interindividual differences in self-reported happiness were quite stable over a 3-year time period and showed negligible to small mean changes in the total group and MDD and SAD subgroups, except for significant increases in self-reported happiness in the MDD recovery group which had a moderate effect size. Higher levels of happiness predicted recovery from MDD but not SAD, non-occurrence of MDD and SAD in participants without MDD or SAD at baseline, as well as a reduction of depression and anxiety symptom severity 3 years later. Conversely, presence of MDD and SAD, as well as higher depression and anxiety symptom severity predicted a reduction of self-reported happiness 3 years later. Moreover, changes in self-reported happiness were associated with changes in diagnostic status and changes in symptom severity, in particular with depression and depression severity.

So, as expected, self-reported happiness did not show absolute stability but interindividual differences proved to be quite stable over a three-year period even when happiness declined in the case of occurrence of emotional disorders and increased in the case of recovery of emotional disorders. Moreover, effect sizes of the associations of happiness with psychiatric disorders and their symptom severity were negligible to small except for the prediction of the occurrence of MDD and SAD by self-reported happiness and the association of changes in self-reported happiness with changes in depression severity and with recovery of MDD. Of note is that the predictive power of self-reported happiness for the occurrence of depressive and anxiety disorder was attenuated but remained significant by controlling for symptom severity, although effect sizes became small. Our results suggest negligible to small reciprocal prospective relationships between self-reported happiness and psychiatric disorder and symptom severity, and moderately strong relationships between changes of self-reported happiness with recovery from MDD and concurrent changes in depression severity.

These findings are generally consistent with cross-sectional studies showing that overall persons report to be quite happy [24] and that the presence of mental disorders or higher levels of psychiatric disorders and their symptom severity are associated with lower levels of self-reported happiness [8], although subjective levels of happiness remain relatively high in the face of mental health problems. Expanding these cross-sectional findings, our clinical prospective cohort study suggests that not only external life events but also the occurrence and recovery of mental disorders are related to changes in the level of self-reported happiness. These findings are consistent with a birth cohort study in young adults also showing the presence of a reciprocal association between life satisfaction and mental health problems [25].

An interesting question for further research is to what extent persons habituate to mental health problems. Although the available evidence indeed shows that happiness levels are moderately stable over time, this stability however does not preclude large and lasting changes [26]. A growing number of studies suggest that happiness levels do change, that adaptation is not inevitable, and that life events such as divorce, widowhood and unemployment may have long lasting effects on the level of subjective happiness [27, 28]. The availability of only two measurement moments in the present study precludes conclusions about habituation to mental health problems and possible long-lasting scarring effects of remitted mental health problems on subjective happiness. In addition, because many persons had or had had emotional disorders reliable data about the level of happiness before the first incidence of emotional disorders are lacking.

The association of changes in happiness with MDD and depression severity seems more pronounced than with SAD or anxiety severity, as happiness did not predict recovery of SAD, changes in happiness were not associated with recovery from SAD and also temporal relationships with anxiety severity tended to be weaker. This concurs with PET and fMRI studies of happiness using autobiographical recall methods showing that happiness is primarily associated with the activation of anterior cingulate cortex, prefrontal cortex, and insula, brain areas which are also connected to basic emotions such as sadness. However, neuroimaging studies comparing happiness with fear are almost absent [29].

As in previous studies depression was the strongest correlate of happiness [30–32]. The pronounced association of happiness with depressive disorder and depression severity poses the question whether happiness/wellbeing and mental illness constitute opposite ends of the same scale or can be better conceptualized as two (partly) independent although strongly correlated dimensions. The high temporal stability of happiness is frequently mentioned as evidence for a stable characteristic of the person fluctuating around a fixed set point. However as also has been shown on the basis of NESDA data, the 9-year stability over time of symptoms of affective disorders is also relatively high [33] and this could mean that past a certain age, level of psychiatric disorders and their symptom severity is relatively set [34], which is in line with a recent study on the course of affective disorders revealing that chronicity is more the rule than the exception [35].

Strengths of the present study include the large sample size, the use of a clinically representative longitudinal cohort design executed at multiple study sites, and the use of standardized diagnostic assessment procedures. However, some limitations of the study should also be mentioned. (a) We only measured happiness and not general life satisfaction as a more cognitive evaluation of the quality of one’s life experiences [4]. Moreover, we only measured subjective well-being from a hedonistic perspective. Another approach to happiness and well-being focuses on meaning and self-realization and defines well-being in terms of the degree to which a person is optimally functioning [36]. According to a eudaimonic approach, individuals are considered to be psychologically healthy when they are fully functioning in their personal life. fMRI studies examining the neural correlates of hedonic and eudaimonic happiness show that both activate a network involving frontal, temporal and parietal regions, as well as subcortical structures. However, hedonic happiness results in enhanced activity in frontal medial/middle regions and anterior cingulate cortex, while eudaimonic happiness in increased activity in the right precentral gyrus [37]. Future (neuroimaging) studies from a eudaimonic perspective examining whether measurements of happiness also strongly covary with indices of psychiatric disorders and their symptom severity are warranted. (b) Only two assessment points were used in this study because self-reported happiness was only measured at these points in the NESDA study precluding conclusions about causality and the duration of the effects on happiness. (c) Notwithstanding the relatively large sample, the size of the occurrence, recovery, and chronically affected MDD and SAD subgroups did not allow to differentiate between pure depression or SAD disorder and comorbid depression and SAD with sufficient statistical power. In addition, other anxiety diagnoses included in NESDA (i.e., panic disorder with or without agoraphobia, generalized anxiety disorder, and agoraphobia without panic) were not further analyzed. The prevalence rates of these other anxiety disorders were too low to create occurrence, recovery and chronically affected subgroups of sufficient size in order to analyze between group difference with enough statistical power. (d) The findings may not be representative of all affective disorders as people with e.g. a bipolar disorder or an obsessive-compulsive disorder were excluded from the sample. Also, participants with a primary diagnosis of severe psychiatric disorders such as a psychotic disorder or severe addiction disorder were excluded in NESDA. Future research into a wider array of disorders is warranted to examine the generalizability of the present study findings across disorders. (d) Results may be subject to social desirability and response bias because of the use of a single item instruments for self-reported happiness in our study. (e) Selective attrition of participants with higher levels of psychiatric disorders and their symptom severity may have confounded the magnitude of the differences in happiness and psychiatric disorders and their symptom severity over time, but will not have critically affected the main analyses of cross-sectional and prospective relationships between these variables. (f) The relatively high associations of self-reported happiness with self-reported symptom severity may be partly due to same method variance. However, given the found associations of self-reported happiness with observer-based psychiatric diagnoses, temporal relations of happiness with psychiatric disorder cannot be totally accounted for by this measurement artifact. (g) When collecting the data for the present study with the CIDI according to DSM-IV criteria the DSM-IV was still widely used. However, as the diagnostic criteria for MDD as well as the essential features of social anxiety disorder have remained the same in DSM 5, study results concerning these specific diagnoses still seem applicable to MDD and SAD according to DSM 5 criteria.

## Conclusion

We found that notwithstanding rather stable interindividual differences in self-reported happiness changes in psychiatric disorder and symptom severity and in particular depression (severity) show moderately strong relations with changes in self-reported subjective happiness. Future studies are needed to examine whether the same applies to measurements of broader concepts of positive well-being in terms of the degree to which a person is optimally functioning. Targeted intervention may be needed to promote positive health and realize optimal functioning after recovery form mental disorders, although persons may feel subjectively happier after recovery.

## Data Availability

According to European law (GDPR) data containing potentially identifying or sensitive patient information are restricted; our data involving clinical participants are not freely available in a public repository. However, data are – under some specifications - available upon request via the NESDA Data Access Committee (nesda@ggzingeest.nl). See also our website: www.nesda.nl

## Abbreviations

NESDA: Netherlands Study of Depression and Anxiety
SRH: Self-Rated Happiness scale
CIDI: Composite Interview Diagnostic Instrument
IDS: Inventory of Depressive Symptomatology
BAI: Beck Anxiety Inventory
FQ: Fear Questionnaire
MDD: Major depressive disorder
SAD: Social anxiety disorders

## References

[1] Prince M, Patel V, Saxena S, Maj M, Maselko J, Phillips MR, Rahman A (2007). No health without mental health. Lancet..

[2] Ryan RM, Deci EL (2001). On happiness and human potentials: a review of research on hedonic and eudaimonic well-being. Annu Rev Psychol.

[3] Diener E (2013). The remarkable changes in the science of subjective well-being. Perspect Psychol Sc.

[4] DeNeve KM, Cooper H (1998). The happy personality: a meta-analysis of 137 personality traits and subjective well-being. Psychol Bull.

[5] Lykken D (1999). Happiness: what studies on twins show us about nature, nurture, and the happiness set-point.

[6] Headey B, Wearing A (1992). Understanding happiness: a theory of subjective well-being.

[7] Brickman P, Coates D, Janoff-Bulman R (1978). Lottery winners and accident victims: is happiness relative?. J Pers Soc Psychol.

[8] Layard R, Chisholm D, Patel VL, Saxena S, Helliwell J, Layard R, Sachs J (2013). Mental illness and unhappiness. World happiness report 2013.

[9] Spinhoven P, Elzinga BM, Giltay E, Penninx B. Anxious or depressed and still happy? PLoS One. 2015;10(10). 10.1371/journal.pone.0139912. 10.1371/journal.pone.0139912 PMC4603679 26461261

[10] Penninx BW, Beekman AT, Smit JH, Zitman FG, Nolen WA, Spinhoven P (2008). The Netherlands Study of Depression and Anxiety (NESDA): rationale, objectives and methods. Int J Methods Psychiatr Res.

[11] Veenhoven R (1995). The cross-national pattern of happiness: test of predictions implied in three theories of happiness. Soc Indic Res.

[12] Abdel-Khalek AM (2006). Measuring happiness with a single-item scale. Soc Behav Pers.

[13] American Psychological Association (1994). Diagnostic and statistical manual of mental disorders.

[14] Wittchen HU (1994). Reliability and validity studies of the WHO Composite International Diagnostic Interview (CIDI): a critical review. J Psychiatr Res.

[15] Rush AJ, Giles DE, Schlesser MA, Fulton CL, Weissenburger J, Burns C (1986). The Inventory for Depressive Symptomatology (IDS): preliminary findings. Psychiatry Res.

[16] Rush AJ, Gullion CM, Basco MR, Jarrett RB, Trivedi MH (1996). The Inventory of Depressive Symptomatology (IDS): psychometric properties. Psychol Med.

[17] Beck AT, Brown G, Epstein N, Steer RA (1988). An inventory for measuring clinical anxiety: psychometric properties. J Consult Clin Psychol.

[18] Osman A, Hoffman J, Barrios FX, Kopper BA, Breitenstein JL, Hahn SK (2002). Factor structure, reliability, and validity of the Beck anxiety inventory in adolescent psychiatric inpatients. J Clin Psychol.

[19] Marks IM, Mathews AM (1979). Brief standard self-rating for phobic atients. Behav Res Ther.

[20] Van Zuuren FJ (1988). The fear questionnaire: some data on validity, reliability and layout. Br J Psychiatry.

[21] Cohen J (1988). Statistical power analysis for the behavioral sciences.

[22] Cicchetti DV (1994). Guidelines, criteria, and rules of thumb for evaluating normed and standardized assessment instruments in psychology. Psychol Assess.

[23] IBM Corporation (2019). SPSS statistics for windows, version 26.0.

[24] Diener E, Diener C (1996). Most people are happy. Psychol Sc.

[25] Fergusson DM, McLeod GF, Horwood LJ, Swain NR, Chapple S, Poulton R (2015). Life satisfaction and mental health problems (18 to 35 years). Psychol Med.

[26] Lucas RE (2007). Adaptation and the set-point model of subjective well-being: does happiness change after major life events?. Curr Dir Psychol Sc.

[27] Lucas RE (2005). Time does not heal all wounds. A longitudinal study of reaction and adaptation to divorce. Psychol Sc.

[28] Diener E, Diener C, Choi H, Oishi S (2018). Revisiting “Most people are happy”-and discovering when they are not. Perspect Psychol Sci.

[29] Angelo Suardi A, Sotgiu I, Costa T, Cauda F, Rusconi M (2016). The neural correlates of happiness: a review of PET and fMRI studies using autobiographical recall methods. Cogn Affect Behav Neurosci.

[30] Dear K, Henderson S, Korten A (2002). Well-being in Australia--findings from the national survey of mental health and well-being. Soc Psychiatry Psychiatr Epidemiol.

[31] Koivumaa-Honkanen H, Kaprio J, Honkanen R, Viinamaki H, Koskenvuo M (2004). Life satisfaction and depression in a 15-year follow-up of healthy adults. Soc Psychiatry Psychiatr Epidemiol.

[32] Koivumaa-Honkanen HT, Viinamaki H, Honkanen R, Tanskanen A, Antikainen R, Niskanen L (1996). Correlates of life satisfaction among psychiatric patients. Acta Psychiatr Scand.

[33] Struijs SY, Lamers F, Verdam MGE, van Ballegooijen W, Spinhoven P, van der Does W, Penninx BWJH (2020). Temporal stability of symptoms of affective disorders, cognitive vulnerability and personality over time. J Affect Disord.

[34] Caspi A, Moffitt TE (2018). All for one and one for all: mental disorders in one dimension. Am J Psychiatry.

[35] Verduijn J, Verhoeven JE, Milaneschi Y, Schoevers RA, van Hemert AM, Beekman ATF (2017). Reconsidering the prognosis of major depressive disorder across diagnostic boundaries: full recovery is the exception rather than the rule. BMC Med.

[36] Waterman AS (1993). Two conceptions of happiness: contrasts of personal expressiveness (eudaimonia) and hedonic enjoyment. J Pers Psychol.

[37] Costa T, Suardi A, Diano M, Cauda F, Duca S, Rusconi M, Sotgiu I (2019). The neural correlates of hedonic and eudaimonic happiness: an fMRI study. Neurosci Lett.

